# Influence of stress distribution in coal seams of non-uniform extremely thick key stratum and disaster-causing mechanisms

**DOI:** 10.1038/s41598-022-18641-7

**Published:** 2022-08-24

**Authors:** Shan Ning, Weibing Zhu, Jianlin Xie, Shikang Song, Xiaozhen Wang, Dan Yu, Jinfu Lou, Jialin Xu

**Affiliations:** 1 State Key Laboratory of Coal Resources and Safe Mining, China University of Mining and Technology, Xuzhou, 221116 China; 2 School of Mines, China University of Mining and Technology, Xuzhou, 221116 China; 3 Shaanxi Zhengtong Coal Industry Co., Ltd., Xianyang, 713600 China; 4 Coal Mining Research Institute, China Coal Technology and Engineering Group Co., Ltd., Beijing, 100013 China

**Keywords:** Environmental sciences, Natural hazards, Engineering

## Abstract

This paper analyzes the influence of the overlying extremely thick primary key stratum on the strong mine pressure hazard at the large mining face in Gaojiapu coal mine. The analysis of the distribution characteristics of the primary key stratum in the Gaojiapu coal mine reveals the bow-shaped structural characteristics of the overlying thick primary key stratum. An elastodynamic model was developed using the variational method to calculate and analyze the influence of the movement of the primary key stratum on the stress and energy of the underlying weak rock. The results show that the arch structure of the overlying extremely thick primary key stratum can significantly affect the distribution pattern of stress and strain energy in the coal body, and the stress and strain energy in the coal body are transferred to the middle of the coal column, and the middle region of the coal column enters a high stress state. These results suggest that the change in thickness of the overlying primary key stratum at Gaojiapu in the coal column area is a major factor in the frequent occurrence of impact ground pressure events at the mine. This study explains the causes of frequent impact ground pressure in the lower coal rock mass of the extremely thick primary key stratum, and provides a reference for the prevention and control of impact hazards in the extremely thick primary key stratum.

## Introduction

The long-term and heavy demand for coal resources in China has caused coal mining depths to increase at a rate of 10–25 m per year^1–3^. As the mining depth increases, the complexity of the geology and mining conditions increases^4,5^, and ground pressure problems in stopes become more prominent. At the same time, the improvement in mining technology and supporting equipment has gradually improved the mining height. This has strengthened the ground pressure appearance in thick seam stopes, and coal or rock dynamic disasters are more frequent^6,7^. It has been observed from many newly built coal mines and production coal mines in western China that during the mining of thick seams, the support frequently shrinks or is crushed resulting in roadway floor heave and rock bursts^8–10^. This phenomenon is caused by the primary key stratum (PKS) above the coal seam being thicker and harder, which greatly changes the PKS movement and the surrounding rock stress distribution^11–13^.

The ground pressure appearance is significantly associated with the movement of the overlying strata^14,15^. Existing research on ground pressure control mainly focuses on the breaking movement of the immediate roof and the first key stratum (the main roof). With the popularization and application of the 3.5–6.0 m fully mechanized mining with large mining height, it is generally recognized that the movement of the key stratum far away from the coal seam will affect the mining pressure of the working face^16,17^. However, the mining of thick seams has large mining space and a wide range of overburden cracks. Thus, it is necessary to consider the influence of the structure and movement of the entire overlying strata.

According to research on Qianqiu coal mine^18–20^, Tongxin coal mine^21,22^, Buertai coal mine^1,23–26^, and other mines in China, thick and hard strata have a large impact on rock mass stress around the working face^27,28^. The surrounding rock stress increases rapidly, which leads to rock bursts frequently occurring on the working face and roadways^29,30^. According to China’s statistical data, roof accidents have the highest rates of all coal mining accidents, which seriously threatens the safety and health of employees^31,32^. Therefore, roof accidents should become a key point in coal mining accident control^33^.

The existence of extremely thick PKS causes a series of damages, which are contrary to the traditional knowledge. From research on the Haizi Coal Mine^34,35^, the existence of extremely thick igneous rock PKS will cause coal and gas burst^36,37^. At the same time, the stress superposition of the thick and hard strata and the strip coal pillar can easily induce rock burst disasters^25,38,39^. Previous studies using simulations have shown that thick and hard strata will change the stress and strain energy transfer mechanism, leading to instability of the roadway^40^.

It is particularly important to master the influence of the extremely thick PKS on the rock mass stress, and to prevent and eliminate the danger of rock burst^41^. So far, studies have not focused on the extremely thick PKS movement and its influence on the surrounding rock stress. Existing research regards PKS as a uniformly thick rock stratum, ignoring its thickness variation. Hence, it is difficult to explain the difference in the influence of extremely thick PKS on the rock mass stress in different regions. Overall, these studies cannot accurately explain the stress transfer path of extremely thick PKS, and there are few studies on energy distribution and failure characteristics of the rock mass under this situation.

Based on the key stratum theory, this study analyzes the PKS occurrence characteristics of Gaojiapu coal mine, and proposes the arch structure of an extremely thick PKS. With regards to this feature, variational methods are used to establish the elasticity mechanics model of nonuniform thickness PKS. Through calculation and analysis, it is clear that the arch structure affects the stress and energy of the coal pillar. Furthermore, the influence of the extremely thick PKS arch structure on the stress distribution of the main entry pillar during the mining process is examined using the universal distinct element code (UDEC). The present work is the first systematic investigation into the influence of PKS occurrence characteristics (thickness and distance from the coal seam). It explores the influence of the PKS thickness variation on stress and energy distribution, and explains the cause of rock burst in the main roadway of the No. 1 panel, thereby providing important reference for improving and enriching ground pressure and ground control, while avoiding rock burst and realizing safe and efficient mining of thick coal seams.

## Background

### Distribution of rock burst events in Gaojiapu coal mine

**Figure 1:**
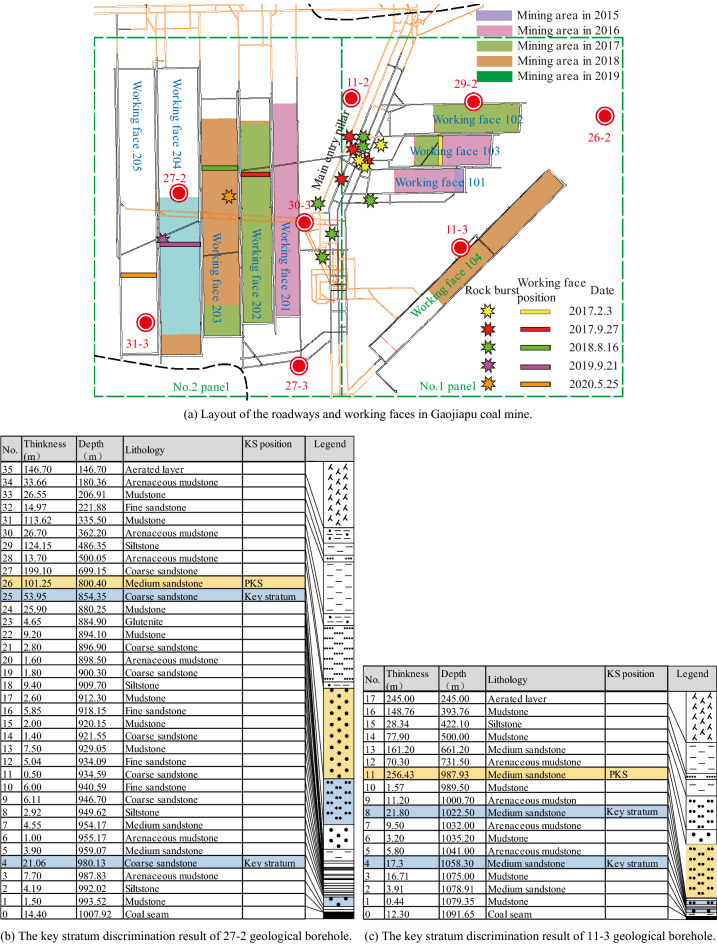
Layout of the working faces and the key stratum discrimination results.

The Gaojiapu coal mine is located at Changwu County, Shaanxi Province. It is a newly built modern mine with a production capacity of 5 Mt/a. Most of the coal seams in this mine have a buried depth of 800–1000 m, with a maximum buried depth of 1076 m, which is a typical deep mine. The coal seam is relatively stable, with an average thickness of 10.5 m and a maximum thickness of 15.75 m. The mine adopted a vertical shaft development method, and No. 1 panel was the first mining panel of the mine (Fig. 1). The 101 working face is the first comprehensive mechanized caving coal mining working face of the mine, where mining started in December 2015.

The 4 seams of the Gaojiapu coal mine have been identified as having a strong propensity to collide, with multiple power events occurring during the excavation of the three main roads in No.1 panel area and frequent impact ground pressure power manifestations occurring during the construction of the mine. Since 2017, the mine has experienced multiple rock bursts, and they are concentrated in the main roadway in No. 1 panel. During the early mining of the 101 and 201 working faces, there was no rock burst event; when the 103 working face was mined, the first rock burst occurred (on 3rd February 2017). Subsequently, the 202 and 203 working faces were mined and rock burst occurred twice (on 27th September 2017 and 16th August 2018). The locations of the first three rock bursts were relatively concentrated in the central area of the main entry pillar in No. 1 panel, which was far away from the working face. When mining the subsequent 204 and 205 working faces, the two rock bursts occurred in the goaf area of the 203 and 204 working faces. As it is located in the goaf, it has a minor effect on mining production.

According to statistical data (Table 1), the rock bursts were concentrated at the top of the main entry pillar in No. 1 panel, and a small number of incidents occurred in the coal seam and floor. According to Table 1, it can be seen that rock bursts mainly occur in the range of 20–85 m above the coal seam. This indicates that the main gate pillar in No. 1 panel is in a high-stress state as a whole, and there is a certain risk of rock burst.

**Table 1:** Rock burst statistics.

Date	Time	Location	Energy (J)
2017/2/3	15:23:33	28 m above the coal seam	7.3 × 10^5^
2017/2/3	15:46:53	38 m above the coal seam	1.4 × 10^5^
2017/2/3	16:03:46	36 m above the coal seam	2.5 × 10^5^
2017/9/27	16:27:43	85 m above the coal seam	1.9 × 10^5^
2017/9/27	20:16:30	3 m below the coal seam	2.9 × 10^5^
2017/9/28	5:55:37	41 m above the coal seam	8.1 × 10^4^
2017/9/28	21:58:31	20 m below the coal seam	1.9 × 10^5^
2018/8/15	11:41:40	62 m above the coal seam	8.8 × 10^5^
2018/8/16	18:32:50	37 m above the coal seam	1.2 × 10^5^
2018/8/16	20:06:06	27 m above the coal seam	8.8 × 10^6^
2018/8/17	7:37:44	50 m above the coal seam	3.7 × 10^4^
2018/8/17	14:15:45	43 m above the coal seam	6.2 × 10^4^
2018/8/19	11:08:04	Coal seam	1.4 × 10^5^

At the same time, as the mining area increases, the area where rock bursts occur gradually increases. In some areas, rock bursts appeared multiple times. This suggests that as mining progresses, the abutment pressure continues to grow, and the area of the highly stressed zone increases.

### Thickness and distance from the coal seam of PKS

**Figure 2:**
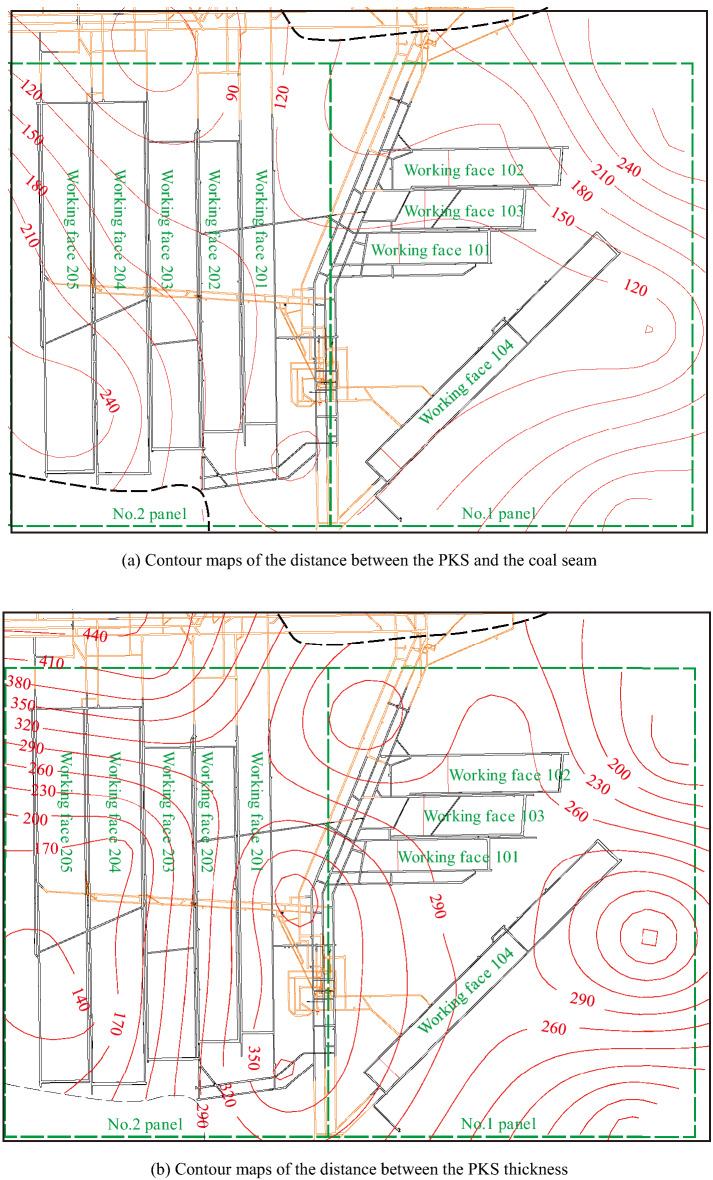
Contour maps of key stratum discrimination results.

Understanding the distribution law of the PKS overlying the working face is important for analyzing the causes of rock bursts in the mine^42,43^. A total of 27 geological boreholes in the Gaojiapu coal mine were analyzed, and some of the key stratum discrimination results are shown in Fig. 1b,c. The KSPB software was used to perform calculations for the key strata. The thickness and spacing of the PKS were determined. Surfer software was used to generate contour maps of the distance between the PKS and the coal seam and thickness contour maps (shown in Fig. 2). With its powerful interpolation and mapping capabilities, Surfer is a popular professional mapping software for geologists.

The analysis of the distance between the PKS and the coal seam (Fig. 2a) shows that the distance between the PKS and the coal seam in panels 1 and 2 of the Gaojiapu coal mine varies greatly, ranging from 110 to 240 m. In panels 1 and 2, the distance between the PKS and coal seam is relatively large, i.e., between 110–180 and 110–240 m, respectively. Among them, the minimum distance between the coal seam and the PKS is 110 m, which is located at the main entry pillar in No. 1 panel. The distance between the coal seam and the PKS is small at the main entry pillar and large in the goaf area on both sides.

The analysis of PKS thickness (shown in Fig. 2b) shows that the PKS thickness is the largest at the top part of the main entry pillar in No. 1 panel, and the PKS thickness at the upper part of the goaf in panels No. 1 and No. 2 is relatively small. The PKS thickness above the goaf in No. 1 panel is between 260 and 290 m; the PKS thickness above the goaf in No. 2 panel is between 140 and 350 m. At the main entry pillar in No. 1 panel, the PKS thickness was distributed in the range of 260–380 m. The PKS thickness is large at the main entry pillar in No. 1 panel and small at the goaf area on both sides.

### Deformation and surface subsidence characteristics of extremely thick PKS

**Figure 3:**
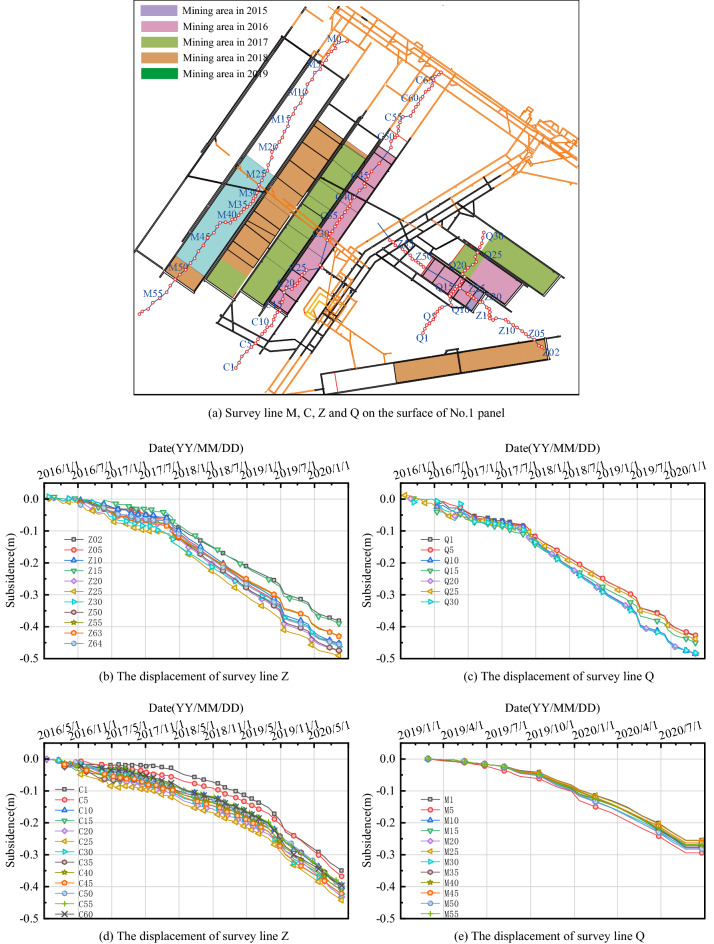
Survey line distribution and settlement curves of some survey points of Gaojiapu coal mine.

According to the general subsidence law, a critical full state has been reached under this mining size and theoretically subsidence should be significant (shown in Fig. 3). According to the results of the current subsidence measurements, the maximum subsidence monitored by the strike line of each working face is almost always around 400 mm, and the same overall subsidence characteristics exist at the measurement points along the entire line. The maximum subsidence value in the No.2 panel area is only 443 mm under mining thickness of about 10 m and burial depth of 950–1000 m, and its response subsidence coefficient is only 0.044, while the subsidence coefficient may reach 0.5–0.8 under normal circumstances. At the same time, moving boundaries are monitored on the strike and tendency lines, which do not reflect the characteristics of the subsidence basin. The overlying rocks did not sink sufficiently after the coal seam was mined in the No.2 panel area and the overlying rocks did not break up.

### PKS arch structure of Gaojiapu coal mine

**Figure 4:**
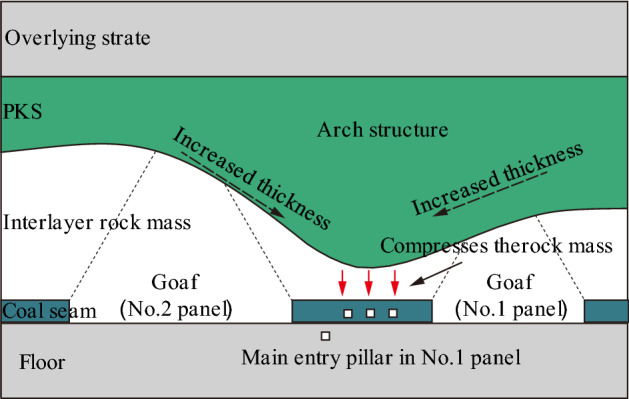
PKS arch structure of the main entry pillar at the No. 1 panel.

From the analysis of the PKS, it is revealed that the thickness of the PKS at the main entry pillar at the No. 1 panel is relatively large, and the distance between the PKS and coal seam is relatively small. The thickness of the PKS in the goaf on both sides is small, and the distance between the PKS and coal seam is relatively large. Meanwhile, according to geological data, the extremely thick sandstone layers to which the PKS belongs are all the Lower Cretaceous Luohe Formation. The upper boundary of the Luohe Formation is buried between 550 and 650 m, with relatively little variation. Therefore, the PKS forms an obvious arch structure on the upper part of the main entry pillar at the No. 1 panel (Fig. 4).

As shown in Fig. 4, the PKS arch structure is located in the upper part of the coal pillar. Under the action of the upper load, the PKS subsides as a whole and compress the interbedded rock mass. Due to the influence of the PKS arch structure, the degree of compression varies in different areas of the rock mass. In the middle of the coal pillar, due to the large protruding area of the arch structure, the thickness of the interbedded rock is smaller and the compression degree is greater under the influence of the overlying PKS. At the edge of the coal pillar, the rock is less compressed due to the greater thickness of the interbedded rock. When the interbedded rock is more compressed, the internal stresses and strains in the rock mass are greater and the elastic energy accumulated in the rock mass is greater. This leads to a high stress and high energy state in the middle of the coal column for a long time. As the No. 1 panel is located in a high stress and high energy area in the middle of the coal column, this makes the main entry very susceptible to damage. Due to the huge energy released during the destruction of the rock mass, several severe rock bursts have occurred in this area.

## Mechanical model of influence of non-uniform thickness PKS on lower rock mass

### Mechanical model of non-uniform extremely thick PKS

**Figure 5:**
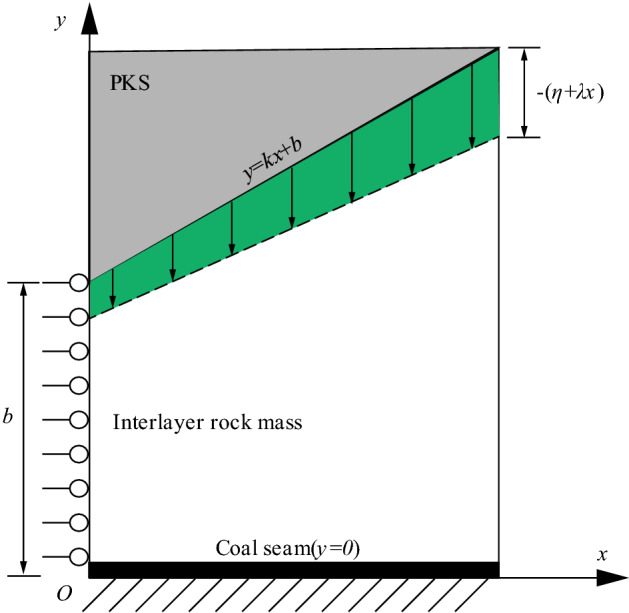
The elastic mechanic model of PKS arch structure.

To investigate the influence of the extremely thick PKS on coal and rock mass stress in the main entry pillar, the elastic mechanics variation method was used to calculate the rock stress. To simplify the calculation, only the area on one side of the central axis of the pillar was considered, and it is simplified to the model shown in Fig. 5. Here, the PKS is regarded as a rigid body that does not deform under an upper load. The bow area of the PKS is simplified to a straight line, the bottom slope change is simplified to *k*, and the distance between the PKS and coal seam is *b*. The main entry pillars are regarded as a whole and deformed under the compression of the PKS. The top boundary satisfies the relationship *y* = *kx* + *b*, where *k* is the slope of change between the PKS and coal seam, and* b* is the minimum distance between the PKS and coal seam. The left boundary is *x* = 0, the bottom boundary is *y* = 0, and the right boundary is *x* = *a*.

The stress change at the main entry pillar is primarily affected by the movement of the overlying extremely thick PKS; so, the displacement of the PKS after coal mining is used as the boundary condition. When the displacement boundary condition is adopted, the stress in the rock mass is related to the boundary displacement. When the displacement was 0, the stress in the coal and rock mass was 0. Therefore, the estimated result is the change in stress caused by the squeezing of the rock after the displacement of the PKS.

As the PKS is extremely hard, only vertical displacement is generated during the subsidence stage whereas no horizontal displacement is generated. Considering the subtle differences in the subsidence values of each part of the PKS, the displacement of the top boundary is given in Eq. (1).

1 $$\left\{\begin{array}{l}{\left({\sigma }_{y}\right)}_{x=0}=\frac{-E\eta }{b}\frac{\left(1-\mu \right)}{\left(1-2\mu \right)\left(1+\mu \right)}\\ {\left({\sigma }_{y}\right)}_{x=250}=\frac{-E\left(\eta +250\lambda \right)\left(1-\mu \right)}{\left(b+250k\right)\left(1-2\mu \right)\left(1+\mu \right)}\end{array},\right.$$

where *u* is the PKS horizontal displacement (m), *v* is the PKS vertical displacement (m), *η* is the overall PKS sinking distance (m), and *λ* is the PKS slope of subsidence in different areas.

The left boundary of the model is the central axis of the coal pillar. During the upper compression process, no horizontal displacement occurred, and only vertical displacement took place. Therefore, the left boundary was set as a simply supported boundary. The lower boundary was the coal seam, and the coal seam floor was set as a fixed boundary without deformation. The right boundary is a mined area, and it is regarded as a free edge; so, the displacement of the right boundary is not considered.

### Mechanical model solution

The variational method^44^ was used to solve the problem, and the displacement trial function was set as given in Eq. (2).

2 $$\left\{\begin{array}{l}u=A\left(1-\frac{y}{kx+b}\right)\frac{y}{kx+b}\frac{x}{a}\\ v=-\left(\eta +\lambda x\right)\frac{y}{kx+b}+B\left(1-\frac{y}{kx+b}\right)\frac{y}{kx+b}\end{array}.\right.$$

In the formula, *A* and *B* are uncorrelated coefficients. To simplify the calculation, only one undetermined coefficient (*A*_1_, *B*_1_) is taken, which is represented by *A* and *B* in the above formula.

The boundary conditions shall be met on each boundary:

3 $$\left\{\begin{array}{l}{u}_{x=a}=0 \\ {u}_{y=0}=0\\ {u}_{y=kx+b}=0\\ {v}_{y=0}=0\\ {v}_{y=kx+b}=-\eta -\lambda x\end{array}.\right.$$

As there is no stress boundary condition, it is considered that the boundary shown in Eq. (3) satisfies all boundary conditions because it satisfies the only boundary condition. The Galerkin method was used to solve the problem.

This study does not consider the influence of rock mass. Given that *f*_*x*_ = *f*_*y*_ = 0, the Galerkin variational equation is:

4 $$\left\{\begin{array}{l}{\int }_{0}^{a}{\int }_{0}^{kx+b}\frac{E}{2\left(1+\mu \right)}\left(\frac{1}{1-2\mu }\frac{\partial \theta }{\partial x}+{\nabla }^{2}u\right){u}_{1}dxdy=0\\ {\int }_{0}^{a}{\int }_{0}^{kx+b}\frac{E}{2\left(1+\mu \right)}\left(\frac{1}{1-2\mu }\frac{\partial \theta }{\partial y}+{\nabla }^{2}v\right){v}_{1}dxdy=0\end{array}\right.,$$

where *E* is the modulus of elasticity (GPa) and *μ* is Poisson’s ratio.

Equations (2) and (4) can be calculated as *A* and *B*

5 $$\begin{array}{l}\left\{\begin{array}{l}A=\frac{-5ak\left(k\eta -b\lambda \right)\left(ak+b\mathit{log}\left[b\right]-b\mathit{log}\left[b+ak\right]\right)}{ak\left(2b\xi -ak\varphi \right)+2{b}^{2}\xi \mathit{log}\left[b\right]-2{b}^{2}\xi \mathit{log}\left[b+ak\right]}\\ B=0\end{array}\right.\\ \xi =5-4{k}^{2}\left(\mu -1\right)-10\mu \\ \varphi =5-3{k}^{2}\left(\mu -1\right)-10\mu \end{array}.$$

The stress and strain components can be obtained by introducing the Eq. (5) into the Eq. (2) and combining geometric and physical equations. Because the coefficient A is complex and constant, A is still used in the calculation process.

6 $$\left\{\begin{array}{l}{\varepsilon }_{x}=\frac{Ay\left({b}^{2}+bkx-by+kxy\right)}{a{\left(b+kx\right)}^{3}}\\ {\varepsilon }_{y}=-\frac{\eta +x\lambda }{b+kx}\\ {\gamma }_{xy}=\frac{Ax\left(b+kx-2y\right)+ay\left(k\eta -b\lambda \right)}{a{\left(b+kx\right)}^{2}}\end{array},\right.$$

7 $$\left\{\begin{array}{l}{\sigma }_{x}=\frac{E\left(a{\left(b+kx\right)}^{2}\left(\eta +x\lambda \right)\mu +Ay\left({b}^{2}+bkx-by+kxy\right)\left(\mu -1\right)\right)}{a{\left(b+kx\right)}^{3}\left(1+\mu \right)\left(-1+2\mu \right)}\\ {\sigma }_{y}=\frac{E\left(Ay\left({b}^{2}+bkx-by+kxy\right)\mu +a{\left(b+kx\right)}^{2}\left(\eta +x\lambda \right)\left(\mu -1\right)\right)}{a{\left(b+kx\right)}^{3}\left(1+\mu \right)\left(-1+2\mu \right)}\\ {\tau }_{xy}=\frac{E\left(Ax\left(b+kx-2y\right)+ay\left(k\eta -b\lambda \right)\right)}{2a{\left(b+kx\right)}^{2}\left(1+\mu \right)}\end{array}\right.,$$

where *ε*_*x*_ is the horizontal strain, *ε*_*y*_ is the vertical strain, *γ*_*xy*_ is the shear strain, *σ*_*x*_ is the horizontal stress in MPa, *σ*_y_ is the vertical stress in MPa, and *τ*_*xy*_ is the shear stress (MPa).

### Solution of stress and strain energy

According to the assumption of the elastic mechanics model in this study, the coal seam coordinates are *y* = 0, which is introduced into the Eqs. (6) and (7) obtained in the previous paragraph to calculate the strain and stress of the coal body, and we can obtain:

8 $$\left\{\begin{array}{l}{\sigma }_{x}=\frac{-E\left(\eta +x\lambda \right)\mu }{\left(b+kx\right)\left(1-2\mu \right)\left(1+\mu \right)}\\ {\sigma }_{y}=\frac{-E\left(\eta +x\lambda \right)\left(1-\mu \right)}{\left(b+kx\right)\left(1-2\mu \right)\left(1+\mu \right)}\\ {\tau }_{xy}=\frac{AxE}{2a\left(b+kx\right)\left(1+\mu \right)}\end{array}\right..$$

The strain and stress of the coal mass were calculated, and the strain energy density was calculated. According to elastic mechanics, the two-dimensional plane strain energy density is given by

9 $${\nu }_{\varepsilon }=\frac{1}{2}\left({\sigma }_{x}{\varepsilon }_{x}+{\sigma }_{x}{\varepsilon }_{x}+{\gamma }_{xy}{\tau }_{xy}\right),$$

where *ν*_*x*_ is the strain energy density, J/m^3^.

Substituting Eq. (6) into Eq. (9), and further simplify it to obtain:

10 $${\nu }_{\varepsilon }=\frac{{A}^{2}{x}^{2}E}{2{a}^{2}{\left(b+kx\right)}^{2}\left(1+\mu \right)}+\frac{E{\left(\eta +x\lambda \right)}^{2}\left(1-\mu \right)}{{\left(b+kx\right)}^{2}\left(1-2\mu \right)\left(1+\mu \right)}.$$

## Influence of movement of extremely thick PKS on stress and energy distribution

### Calculation scheme and parameters

With the movement of the PKS, the stress of rock mass changes. With reference to the characteristics of the surface subsidence of Gaojiapu and the displacement of the PKS as a variable, six schemes were designed for comparative analysis (Table 2). Among them, the surface deformation slope is between 1 × 10^–4^ and 2 × 10^–4^, the maximum displacement of the model boundary *u*_*max*_ is between 0.025 and 0.05 m, and the range of the vertical displacement *η* is between 0.02 and 0.4 m.

**Table 2:** Calculation scheme.

Scheme	KS structure		*η*	*λ*	*u*_*max*_
	*b*/m	*k*			
1	100	0.2	0.025	1 × 10^–4^	0.025
2			0.005	1 × 10^–4^	0.025
3			0.100	1 × 10^–4^	0.025
4			0.200	2 × 10^–4^	0.05
5			0.300	2 × 10^–4^	0.05
6			0.400	2 × 10^–4^	0.05

### Influence of extremely thick PKS arch structure on stress

**Figure 6:**
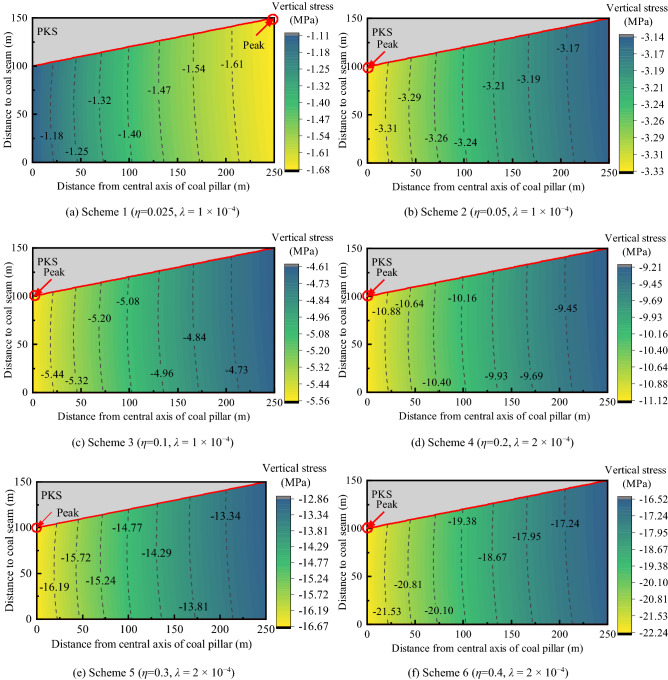
The contour map of the rock mass vertical stress under the PKS arch structure.

Figure 6 is the contour map of the rock mass vertical stress under the PKS arch structure. It shows how the coal and rock mass stress changes with the subsidence of the PKS. The coal and rock mass stress increases gradually with the displacement of PKS. In Fig. 6a, the maximum value of vertical stress is 1.68 MPa and its minimum value is 1.11 MPa; in Fig. 6f, the maximum and minimum values are 22.24 MPa and 16.52 MPa, respectively*.* During this process, the average value of vertical stress increases with the displacement of the PKS, from 1.45 to 18.81 MPa. This result shows that given the effect of the overall subsidence of extremely thick PKS, the increase of coal and rock mass stress is not limited to a certain area, and the overall stress rises.

The coal pillar stress is affected by the PKS arch structure and is concentrated in the central area. The magnitude of the stress change in the middle of the coal pillar is large whereas the edge stress is small. Owing to the relatively large subsidence of the PKS in the early stage of mining (Fig. 6a), the stress at the edge of the pillar is relatively large, i.e., 1.51 times that of the center of the pillar. As the PKS further subsides (Fig. 6b–f), the stress at the center of the pillar is greater than the stress at the edge (it is 1.34 times that of the edge). This indicates that the PKS arch structure will cause higher stress concentration at the center of the main entry pillars.

### Influence of PKS arch structure on energy distribution

**Figure 7:**
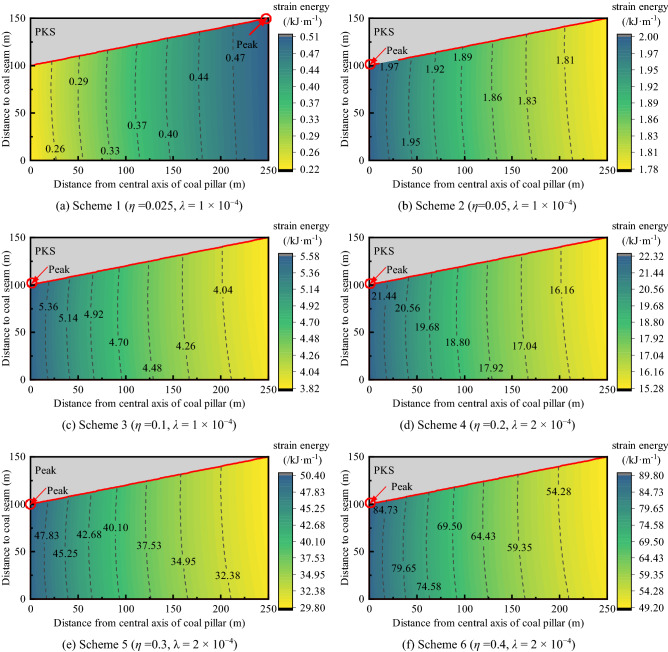
The contour map of the rock mass strain energy under the PKS arch structure.

In the previous section, the influence of the PKS arch structure on the rock mass stress in the main entry pillars was analyzed, and there was a relatively evident stress concentration in this area. Owing to this factor, the strain energy of this region also changes. Figure 7 is a contour plot of the strain energy distribution under the PKS arch structure.

Similar to the distribution of vertical stress, the strain energy in the coal and rock mass stress increased overall as well (Fig. 7). When the displacement of the PKS increases, the maximum strain energy density gradually increases from 0.51 (Fig. 7a) to 89.80 kJ (Fig. 7f). The minimum strain energy density gradually increased from 0.22 (Fig. 7a) to 49.2 kJ (Fig. 7f). During this process, the average strain energy increased from 0.39 to 64.32 kJ.

As the strain energy of the coal pillar increased, the strain energy shifted to the center of the coal pillar. In Fig. 7b, the strain energy density at the center of the coal pillar is approximately 1.12 times that at the edge, and in Fig. 7f, the strain energy density at the center of the coal pillar is approximately 1.82 times that at the edge. This result indicates a greater concentration of the strain energy distribution in the central region that is affected by the arch structure and greater amount of energy released during the fracture process of the rock mass in this region.

### Influence of PKS arch structure on stress distribution of coal seam

**Figure 8:**
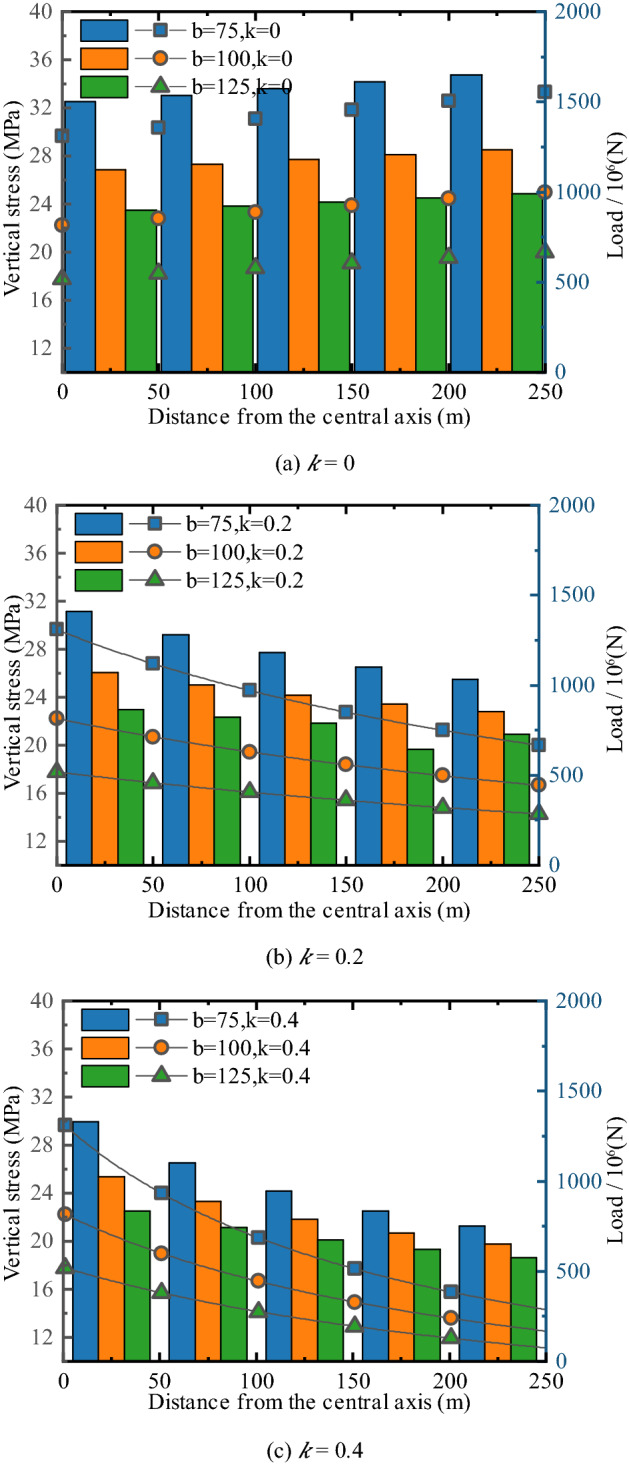
The stress and load distribution in different PKS structures.

To analyze the influence of the extremely thick PKS on the coal body, the stress of the coal body under the PKS with different parameters was analyzed. Since the stress state of the coal seam is affected by the displacement of the PKS, the displacement of the PKS is taken as option 6 in Table 2. At the same time, the distance between the arch structure of the PKS and coal seam is *b*, and the slope of the bottom interface of PKS is *k*. Among them,* b* is equal to 75 m, 100 m, and 125 m, while *k* equals 0, 0.2, and 0.4. When *k* = 0, the PKS is of uniform thickness, and there is no arch structure. The coal seam is divided into five sections at 50 m intervals, and each section is integrated to calculate the load. During the calculation process, the *y* and *z* directions consider the unit length. Concurrently, the absolute value of vertical stress σ_y_ is considered to facilitate the analysis. The results are shown in Fig. 8.

The data in Fig. 8 show that when the PKS does not have an arch structure, the coal mass stress is high at the edge and low in the middle. In Fig. 8a, there is no arch structure when *k* = 0. When* b* = 75 m, the central and edge stresses of the coal pillar are 29.64 MPa and 33.33 MPa, respectively; when* b* = 125 m, the central and edge stresses are 17.778 MPa and 20 MPa, respectively.

When there is an arch structure, the coal mass stress is concentrated at the center of the coal pillar, and the coal mass stress is low at the edge and high in the middle. When* b* = 75 m, the central and edge stress of the coal pillar are 29.63 MPa and 20 MPa, respectively; when* b* = 125 m, the central and edge stress of the coal pillar are 17.778 MPa and 14.286 MPa, respectively. In this case, the middle part of the coal pillar could bear more load. Taking* b* = 75 m as an example, the overburden load in the range of 0–50 m is 1407.7 × 10^6^ N; additionally, the overburden load in the range of 200–250 m is 1030.5 × 10^6^ N. The central part of the coal pillar bears approximately 1.36 times the load of the edge area.

After comparing the data in Fig. 8, it is found that as the thickness of the arch top position increases, the stress concentration at the center of the coal pillar becomes more obvious. When* b* = 125 m and *k* = 0, the PKS is uniform, and the distance between the coal seam is 125 m. Under these conditions, the central and edge stress of the coal pillar are 17.778 MPa and 20 MPa, respectively, and the overburden loads in the range of 0–50 m and 200–250 m are 900 × 10^6^ N and 988.89 × 10^6^ N. When* b* = 75 m and *k* = 0.2, there is an arch structure with a distance of 125 m from the coal seam, and a distance of 75 m between the arc top. In this case, the central and edge stress of the coal pillar are 29.63 MPa and 20 Mpa, respectively; furthermore, the overburden loads in the range of 0–50 m and 200–250 m are 1407.7 × 10^6^ N and 1030.5 × 10^6^ N, respectively. In this process, the stress and load in the central area of the coal pillar increased by 1.67 times; the stress value in the edge area was equal, and the load only increased by 1.04 times.

Substituting *x* = 0 and *x* = 250 into Eq. (7), we obtain Eq. (11), which shows that under the condition of a certain displacement, when the distance between the edge of the coal pillar and the PKS is the same, the stress of the coal mass is equal. The state of the central area of the coal pillar is primarily affected by the spacing *b*. The vertical stress at the center of the coal pillar is directly proportional to the subsidence *η* and inversely proportional to the PKS spacing *b*.

11 $$\left\{\begin{array}{l}{\left({\sigma }_{y}\right)}_{x=0}=\frac{-E\eta }{b}\frac{\left(1-\mu \right)}{\left(1-2\mu \right)\left(1+\mu \right)}\\ {\left({\sigma }_{y}\right)}_{x=250}=\frac{-E\left(\eta +250\lambda \right)\left(1-\mu \right)}{\left(b+250k\right)\left(1-2\mu \right)\left(1+\mu \right)}\end{array}.\right.$$

This result shows that the arch structure of the extremely thick PKS causes the stress distribution change as well as the rock mass in this area to always be at a higher stress level. With an increase in the mining area, the overall sinking distance of the PKS gradually increased. When mining disturbance affects this area, it easily causes damage and releases a large amount of energy.

## Influence of mining on surrounding rock stress

### Numerical model of non-uniform thick PKS

Numerical simulation software is a common tool in the study of mining engineering problems, and UDEC is one of the discrete element programs that are commonly used for discontinuous media problems. The UDEC numerical software is used to simulate the movement and stress distribution of the overburden under different mining conditions, and the movement and stress evolution of the Gaojiapu key stratum studied in this paper.

**Figure 9:**
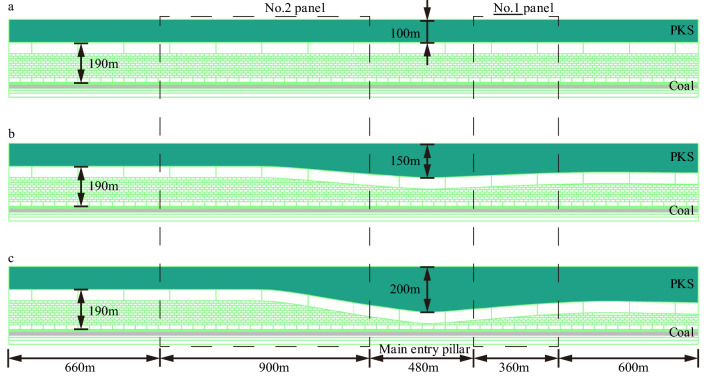
Numerical model of extremely thick PKS.

With regards to the extremely thick PKS arch structure in the Gaojiapu coal mine, a model of the PKS under different thickness changes was established. After changing the maximum thickness of the PKS in the arch structure, the stress change law of coal and rock mass under different conditions was compared and analyzed. Figure 9 shows a two-dimensional numerical model of 3000 m × 340 m. The thickness of the coal seam was 14 m, and the total thickness of the PKS was between 100 and 200 m. One working face (panel) was excavated each time during the mining process of the working face, and the total length of the excavation was 1854 m. The excavation sequence is: No. 1 panel → 201 working face → 202 working face → 203 working face → 204 working face → 205 working face. The numerical simulation test parameters are shown in Table 3.

**Table 3:** Rock mechanics parameters.

Rock stratum	Density (kg m^-3^)	Elasticity modulus (GPa)	Poisson’s ratio	Cohesion (MPa)	Tensile strength (MPa)	Friction angle (°)
PKS	2294	6.31	0.27	23.87	14.77	45
Key stratum	2303	8.71	0.23	23.19	14.33	45
Key stratum	2326	7.59	0.21	23.26	14.03	45
Soft rock	2397	6.30	0.25	23.09	6.34	33
Coal seam	1274	1.99	0.28	19.25	1.17	25

The top boundary of the model was controlled by stress boundary conditions, the upper load was calculated at a buried depth of 500 m, and a vertical stress of 12 MPa was applied. The lateral and bottom boundaries were controlled by the displacement boundary conditions, and horizontal and vertical constraints were applied, respectively. In this numerical simulation, the calculation model of all elements is set as the Mohr–Coulomb elastoplastic model.

### Stress change law of surrounding rock under the influence of mining

**Figure 10:**
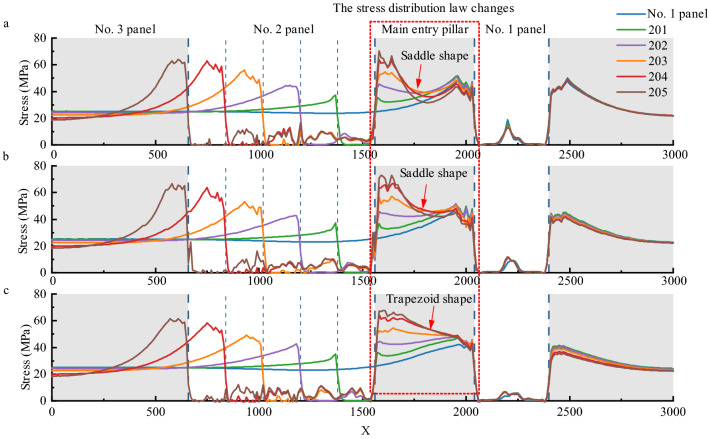
Abutment stress of coal pillars.

According to the results shown in Fig. 10, it can be seen that during the mining process, the abutment stress of coal pillars gradually increases with the mining area, and it is always greater than the stress on both sides of the goaf during the mining process. Taking Option 1 as an example, the peak stress of the coal pillar reaches 70 MPa after the mining is completed, and the peak stress on both sides is 64.19 MPa and 47.85 MPa, respectively. The coal pillar bears more load.

With the change in the PKS thickness, the vertical stress distribution law in this area changed significantly. When panels 1 and 2 were fully mined, the stress distribution of the coal mass changes from a saddle shape to a trapezoid shape. When scheme 1 is adopted, the stress of the coal pillars is large at the edges and small in the middle. The maximum stress at the edge of the coal body in No. 1 and No. 2 panels is 43.8 MPa and 67.5 MPa, respectively, and the stress in the middle of the coal pillar is 31.9 MPa. When scheme 2 is adopted, the stress in the middle of the coal pillar increases significantly, with a stress value of 42.3 MPa, which still presents a saddle-shaped distribution. When scheme 3 is adopted, the stress is 55.4 MPa, and it shows an obvious trapezoidal distribution.

Because of the arch structure, the stress distribution in the coal pillar area changed to a large extent. In comparison with the uniform thickness of the key stratum, when the arch structure exists, the stress in the central area of the coal pillar increases. The arch structure had a significant impact on the stress of the coal pillar.

The stress distribution in the coal pillar area is altered to a large extent by the arch structure. In the presence of arch structure, the stresses in the central part of the coal column increases and the stress distribution in the central part of the coal column shows a trapezoidal distribution. When the key stratum is of uniform thickness, the stress distribution appears saddle-shaped.

### Influence of PKS arch structure on stress distribution law of coal and rock mass

**Figure 11:**
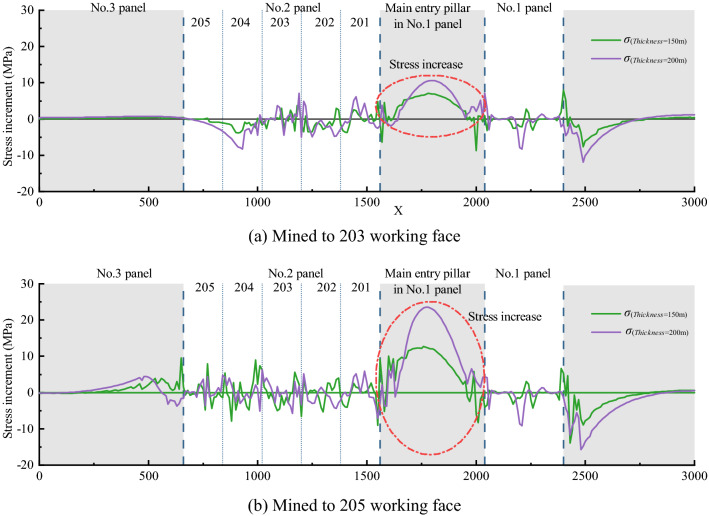
Distribution of stress increment under the condition of changing in thickness of the PKS.

Affected by the arch structure of the PKS, the stress distribution of the coal pillar in the main entry pillar in No. 1 panel changed. In order to compare and analyze the changing situations of surrounding rock stress under different schemes, the stress increments of scheme 2 and scheme 3 relative to scheme 1 are calculated respectively based on scheme 1. Figure 11 shows the stress difference between mining to 203 working face and 205 working face.

The results in Fig. 11 show that the change in thickness of the PKS leads to greater change in the stress at the central area of the coal pillar. When the thickness of the PKS is 150 m, the stress difference in the middle of the coal pillar is 7 MPa when it is mined to the 203 working face, and the stress increment is 12.6 MPa when it is mined to the 205 working face. When the thickness of the PKS is 200 m, the stress increase in the middle of the coal pillar is 10.5 MPa when mining the 203 working face; the stress difference is 23.47 MPa when mining to the 205 working face, which is equivalent to the vertical stress at a depth of 1000 m.

This result shows that the distribution of stress in the middle of the coal pillar changed significantly owing to the change in thickness of the PKS. At the same time, with the increase in the excavation area, the stress increase in this area also increased to a large extent.

### Comparative analysis of theoretical results and numerical simulations

**Figure 12:**
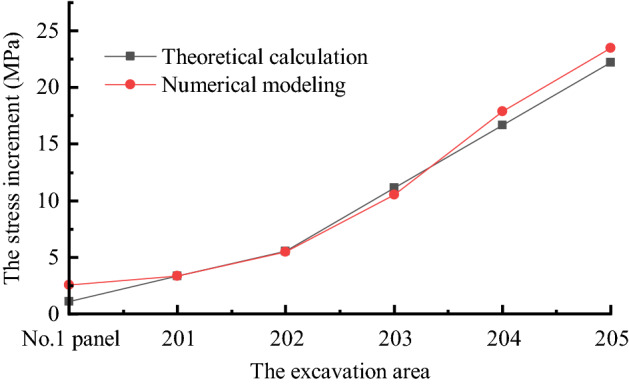
Comparative analysis of theoretical calculation and numerical results.

To verify the accuracy of the theoretical results and numerical simulations, the stress increment values in “Solution of stress and strain energy” section were compared with the stress increment values in “Influence of  PKS arch structure on stress distribution law of coal and rock mass” section. The results obtained are shown in Fig. 12.

As shown in Fig. 12, the stress in the middle of the coal pillar always increases as mining increases. The theoretical results showed a stress increase of 22.2 MPa at the end of the 205 working face, and the numerical simulations showed a stress increase of 23.47 MPa. The two curves remained consistent throughout the process, showing the reliability of the theoretical results and numerical simulations.

## Conclusions

This study reveals the influence of the bow structure of the PKS on mining hazards. The overlying extremely thick PKS and its arch structure in the Gaojiapu coal mine are important reasons for the frequent occurrence of impact ground pressure in the coal pillars of the first panel. Under the overall sinking of the overlying rock layer, the arch structure of the PKS squeezes the rock in the coal pillar area and causes a change in the stress and energy distribution pattern of the rock in this area, forming a high stress concentration area in the central area of the coal pillar, which eventually leads to the occurrence of impact ground pressure.

The main advantage of this study is the detailed analysis of the impact hazard mechanism by means of mechanical analysis and numerical simulation. The stress and strain energy distribution pattern of the coal pillar is altered by the arch structure of the overlying extremely thick PKS at the Gaojiapu Mine. With the sinking of the PKS, the amount of stress change in the middle of the coal column reaches 1.3 times that of the edge, and the stress concentration phenomenon is obvious. The central part of the coal pillar gradually enters a high stress and high energy state.

This study illustrates that an increase in rock thickness in the local area will lead to an increase in stress in the lower coal seam. This suggests that the tectonic characteristics of the overlying coal seam should be considered in the prevention and control of impact hazards. This indicates that attention should be paid to the tectonic features of the overburden rock during impact hazard prevention and control so as to avoid localized stress concentrations caused by specific formations, which could lead to safety accidents.

This research further extends the study of the causes of rock bursts. By studying the specific geological conditions of the mine, it is possible to better develop the mine rock bursts prevention and control plan and determine the high risk areas for rock bursts. The results of this study provide a reference for future production in the mine and can effectively guide the impact pressure prevention and control measures in the subsequent production process of the mine.

## Data Availability

The datasets used and/or analysed during the current study available from the corresponding author on reasonable request.
